# Tuberculose surrénalienne unilatérale isolée

**DOI:** 10.11604/pamj.2015.20.267.6445

**Published:** 2015-03-19

**Authors:** Madiha Mahfoudhi, Hédia Bellali

**Affiliations:** 1 Service de Médecine Interne A, Hôpital Charles Nicolle, Tunis, Tunisie; 2 Service d'Epidémiologie et de Statistiques, Hôpital A, Mami, Ariana, Tunisie

**Keywords:** Surrénale, tuberculose, granulome, nécrose caséeuse, adrenal, tuberculosis, granuloma, caseous necrosis

## Image en medicine

La tuberculose surrénalienne isolée est rare et de diagnostic difficile. Elle est parfois découverte à l'occasion d'une insuffisance surrénalienne. Des cas d'incidentalomes révélant une tuberculose surrénalienne primitive sont exceptionnels. Patient âgé de 42 ans sans antécédents particuliers ni notion de contage tuberculeux, a consulté pour des douleurs de l'hypochondre droit évoluant depuis un mois, une anorexie et un amaigrissement de 7 Kg sans fièvre ni troubles du transit. L'examen physique n'a pas objectivé de masse palpable ni d'hépato-splénomégalie. La tension artérielle était à 130/70 mmHg. L'examen biologique n'a pas révélé de syndrome inflammatoire, ni de signes de malabsorption. L’échographie et la TDM abdomino-pelvienne ont montré une hypertrophie de la surrénale droite sans adénopathies associées. Plusieurs diagnostics ont été évoqués tels qu'un lymphome, une tuberculose ou une néoplasie solide. Le dosage des hormones surrénaliennes était normal. L'intradermo-réaction à la tuberculine était fortement positive. Le Quantiféron test était positif. L'examen anatomopathologique a révélé des granulomes épithéloïdes et giganto-cellulaires avec nécrose caséeuse. Les autres localisations tuberculeuses partculièrement respiratoires, urinaires et digestives ont été éliminées. Le diagnostic de tuberculose surrénalienne droite isolée a été posé. L’évolution était favorable sous un traitement anti-tuberculeux (l'association isoniazide, rifampicine, éthambutol et pyrazinamide pendant deux mois puis l'association isoniazide et rifampicine pendant huit mois) bien conduit.

**Figure 1:**
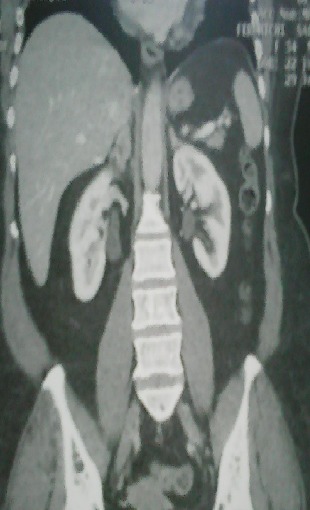
TDM abdomino-pelvienne; hypertrophie surrénalienne droite

